# Artificial Intelligence Enabled Noninvasive Mapping of Cardiac Arrhythmia Origins Using the 12‐Lead Electrocardiogram

**DOI:** 10.1111/jce.70319

**Published:** 2026-03-17

**Authors:** Ibrahim Antoun, Alkassem Alkhayer, Ahmed Abdelrazik, Mahmoud Eldesouky, Kaung Myat Thu, Mokhtar Ibrahim, Riyaz Somani, G. André Ng

**Affiliations:** ^1^ Department of Cardiology University Hospitals of Leicester NHS Trust, Glenfield Hospital Leicester UK; ^2^ Department of Cardiovascular Sciences Clinical Science Wing, University of Leicester Leicester UK; ^3^ Department of Cardiology Guys and St Thomas Hospital London UK; ^4^ National Institute for Health Research Leicester Research Biomedical Centre Leicester UK; ^5^ Leicester British Heart Foundation Centre of Research Excellence Glenfield Hospital, Groby Road Leicester UK

**Keywords:** arrhythmia localisation, artificial intelligence, electrocardiogram, medicine, personalised, ventricular arrhythmias

## Abstract

Artificial intelligence (AI) is reshaping cardiac electrophysiology by extracting information from electrocardiograms that exceeds human visual interpretation. While most AI applications have focused on arrhythmia detection and risk stratification, a newer and more targeted use case is emerging: noninvasive localisation of arrhythmia origins from a standard 12‐lead electrocardiogram (ECG). This review examines the current evidence supporting AI‐driven ECG‐based localisation of arrhythmogenic foci, with an emphasis on accessory pathways and idiopathic ventricular arrhythmias. Recent machine learning and deep learning models have demonstrated high accuracy in predicting anatomical sites of origin. These advances suggest that AI can capture subtle spatiotemporal electrical patterns that correlate with specific cardiac regions, offering a form of virtual pre‐procedural mapping. Early clinical data indicate that AI‐informed localisation may shorten procedure duration, reduce fluoroscopy exposure, and improve ablation efficiency without compromising safety. The review also discusses key technical innovations, including convolutional neural networks, multimodal data integration, and strategies to enhance model generalisability and interpretability. Important challenges remain, particularly around external validation, data quality, clinician trust, and workflow integration. Nevertheless, AI‐driven ECG localisation represents a conceptual shift from diagnostic support toward therapeutic guidance in electrophysiology. As validation studies and translational research advance, this technology has the potential to transform pre‐procedural planning and intraprocedural decision‐making, thereby making arrhythmia ablation more precise, efficient, and accessible.

## 1 Introduction

Artificial intelligence (AI) is rapidly transforming cardiac electrophysiology (EP) by extracting clinically useful information from complex electrical signals that the human eye cannot discern. While early successes focused on automated arrhythmia detection and risk prediction from electrocardiograms (ECGs) [1, 2, 3, 4, 5], newer applications target more nuanced challenges. One emerging niche is the use of AI for noninvasive localisation of arrhythmia sources (ectopic foci or accessory pathways) from 12‐lead ECGs. This goes beyond generic ECG interpretation, aiming to pinpoint the origin of an arrhythmia in the heart, a task traditionally accomplished with invasive catheter mapping during an ablation procedure. Accurate pre‐procedural localisation could streamline EP interventions by informing strategy, reducing mapping time, and potentially improving patient outcomes [6]. In this article, we investigate this underreported application of AI in ECG pre‐procedural localisation, highlighting recent research advances, clinical implications, technical hurdles, and prospects for integrating into EP workflows. The focus is intentionally narrow to provide depth on how AI‐ECG algorithms can assist in identifying arrhythmia origin sites, offering a glimpse of future EP practices that are more precise and personalised.

## 2 AI in ECG‐Based Arrhythmia Localisation

### 2.1 Rationale and Novelty of the Use Case

Localising the origin of arrhythmias (such as focal premature ventricular complexes (PVCs), idiopathic ventricular tachycardias, or accessory pathway conduction in Wolff‐Parkinson‐White syndrome (WPW) is critical for guiding catheter ablation. Traditionally, EP specialists infer likely sites of origin using heuristic ECG algorithms or visual pattern recognition and then confirm these findings with invasive mapping. These manual approaches can be error‐prone and dependent on clinician experience [7]. They also struggle with atypical cases or ambiguous ECG patterns where standard criteria break down [7]. The novelty of AI‐driven localisation lies in its data‐driven pattern recognition. By training on large ECG data sets labelled with confirmed arrhythmia origins (from prior ablation cases), AI models can learn subtle electrocardiographic features that map to specific anatomical sites, potentially outperforming rule‐based algorithms. This approach is particularly valuable in EP, where millisecond differences in activation or slight changes in wave polarity can signify different origin locations. An AI model that reliably interprets these subtleties offers a new level of precision, essentially providing a “virtual EP mapping” from the noninvasive ECG. This is a distinct and underexplored application compared to general arrhythmia classification; it represents a shift from using AI for diagnosis to using AI as a *localisation tool* to guide therapy.

### 2.2 Recent Research Advances

Emerging studies demonstrate the feasibility of AI‐enabled ECG localisation. For example, Nishimori et al. developed a convolutional neural network (CNN) model to identify accessory pathway locations in patients with WPW. In their study, the AI model significantly outperformed a traditional decision‐tree ECG algorithm (used clinically for WPW), achieving roughly 78% localisation accuracy vs. 61% by the conventional method [7]. Moreover, by integrating chest X‐ray data with ECG data (a multimodal deep learning approach), accuracy improved to ~80%, illustrating how anatomical context (heart size/orientation) can enhance predictions. The model could correctly classify pathway location even when the standard algorithm was misled (e.g., a left‐sided pathway misclassified as right‐sided by the rules but correctly localised by the AI). This highlights AI's ability to capture complex, subtle cues that elude simplistic criteria.

Even more striking results have been reported for idiopathic ventricular arrhythmias. Zheng et al. developed a machine‐learning system to predict the origin of PVCs/ventricular tachycardias across 21 distinct anatomical sites using 12‐lead ECG data [8]. With a large training data set (18,000 + ECG recordings from 545 ablation patients), their optimised model achieved ~98% accuracy in identifying the correct site of origin on a held‐out test cohort [8]. This level of precision—exceeding that of prior methods and expert physicians—suggests that AI can discern minute differences in QRS morphologies or timing that correspond to different ventricular regions. Earlier work by Missel et al. and others had demonstrated the potential of hybrid machine learning models for VT localisation with good accuracy [9]. Still, recent advances in deep learning and larger data sets have marked a substantial performance leap. Notably, these models handle hierarchical classification (e.g., distinguishing the right ventricular outflow tract from the left ventricular outflow tract (LVOT) and, at a finer scale, specific cusps or septal locations). They can incorporate multiple beats or leads simultaneously to inform predictions.

**Figure 1:**
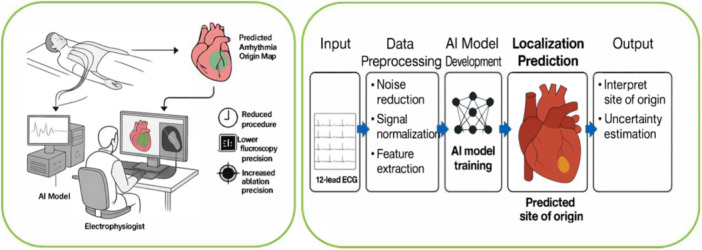
AI‐driven workflow for noninvasive arrhythmia source localisation using 12‐lead ECG.

Beyond retrospective development, there are signs of translation toward real‐world use. Fox et al. reported an “AI‐assisted” arrhythmia mapping workflow in the EP lab, in which insights from 12‐lead ECG analysis guided the initial mapping strategy [6]. In a comparative study, AI‐informed mapping procedures were significantly more efficient, averaging 22.6% shorter in duration and requiring 43.7% less fluoroscopy time without compromising safety or efficacy. In fact, 6‐month arrhythmia‐free survival was slightly higher in the AI‐guided group (73.5% vs. 63.3% in controls). This is an early but powerful example of how AI ECG interpretation can directly impact clinical EP workflows. By localising likely arrhythmia sites noninvasively, the team could target ablation more quickly, reducing patient and operator exposure and possibly improving outcomes. Such findings underscore that AI‐based ECG localisation is not just a theoretical exercise but an emerging reality backed by clinical data. Figure 1 illustrates the proposed AI‐driven workflow for noninvasive arrhythmia source localisation from 12‐lead ECGs, and key studies evaluating AI‐based ECG localisation of arrhythmia origins are summarised in Table 1.

**Table 1:** Selected studies on AI‐Based ECG localisation of arrhythmia origins.

Study (Year)	Arrhythmia type	AI Model type	Localization target	Data set size	Reported accuracy	Clinical impact highlights
Nishimori et al. (2021) [7]	WPW Syndrome	CNN + X‐ray fusion	Accessory pathway (4 zones)	390 patients	78% (AI) vs. 61% (classic)	Improved pre‐ablation planning
Zheng et al. (2022) [8]	PVC/VT	CNN	21 ventricular subregions	18,000+ ECGs (545 pts)	~98%	High‐precision site prediction
Missel et al. (2020) [18]	Idiopathic VT	Machine Learning	RVOT vs. LVOT	282 patients	~88%	Assisted access route planning
Fox et al. (2024) [6]	Mixed (Clinical Use)	AI‐guided Workflow	EP lab integration (real‐time)	191 procedures (case–ctrl)	Not specified	23% faster procedures, 44% less fluoroscopy

Abbreviations: AUC, area under the curve; CNN, convolutional neural network; EP, electrophysiology; LVOT, left ventricular outflow tract; LV, left ventricle; PVC, premature ventricular complex; RVOT, right ventricular outflow tract; VT, ventricular tachycardia; WPW, Wolff–Parkinson–White.

### 2.3 Clinical Implications

The ability to pinpoint arrhythmia origins from a simple ECG has far‐reaching clinical implications for electrophysiology. First, it can significantly enhance pre‐procedural planning. Knowing the probable focus of an arrhythmia before entering the lab allows the electrophysiologist to tailor the strategy. For example, deciding on vascular access routes or mapping catheter selection if a ventricular tachycardia focus is likely in a certain region of the left ventricle versus the right. In WPW patients, accurate localisation of an accessory pathway can guide whether the trans‐septal approach is needed (for left‐sided pathways) or whether mapping can be confined to the right side. AI predictions, being less observer‐dependent, provide a consistent starting point for all operators, potentially levelling the field between less experienced and expert clinicians in terms of mapping success [10].

Second, AI localisation promises shorter and more effective ablation procedures. By highlighting likely arrhythmogenic regions at the outset, the EP team can focus mapping and ablation energy on those targets earlier, as reflected in reduced procedure and fluoroscopy times in the AI‐assisted mapping study [11]. Shorter procedures result in reduced anaesthesia duration and lower X‐ray exposure, thereby improving patient safety. They also translate to lower healthcare costs and higher lab throughput. Importantly, more precise localisation can improve acute success rates (ensuring the true origin is ablated) and possibly long‐term outcomes, as suggested by higher arrhythmia‐free survival in AI‐guided cases. For conditions like PVC‐induced cardiomyopathy, quickly eliminating the culprit PVC focus is crucial; an AI tool that flags the PVC origin might expedite therapy and prevent further ventricular dysfunction.

Another implication concerns diagnostic clarity in complex cases. Certain wide‐QRS tachycardias or ectopic rhythms can be challenging to differentiate (e.g., atypical ventricular tachycardia vs. supraventricular tachycardia with aberrancy). Advanced AI models could classify the mechanism and origin zone, aiding diagnosis when standard criteria are inconclusive. Moreover, AI algorithms provide probability outputs or confidence measures for each predicted site. This allows clinicians to gauge uncertainty: if an ECG does not fit any learned pattern well, the model can indicate low confidence or an “atypical” result, which is informative and can prompt more extensive mapping. AI‐based ECG localisation is most effective in focal arrhythmias with stable activation patterns, such as premature ventricular complexes and accessory pathways. Its performance may be reduced in complex arrhythmias with variable activation sequences, including macro‐reentrant tachycardias or arrhythmias with multiple exit sites, where ECG signatures are less consistent [12].

Finally, AI‐based ECG analysis could broaden access to specialised EP insights. Not every hospital has expert arrhythmia specialists, but an AI tool could assist general cardiologists in identifying patients who require referral for ablation by suggesting a likely arrhythmia type and location [13]. This kind of decision support can act as a “virtual electrophysiologist,” triaging and guiding appropriate care earlier. For example, if a routine clinic ECG is processed through an AI model and indicates a PVC focus in the outflow tract with high confidence, a clinician might more quickly recognise the need for an EP consultation, especially if the patient has symptoms. Thus, from pre‐procedure planning in the EP lab to upstream decision‐making in general practice, the clinical applications of AI‐driven ECG localisation are expansive.

### 2.4 Technical Innovations and Challenges

Implementing AI for arrhythmia source localisation required overcoming several technical challenges, thereby spurring innovative solutions. A key challenge is the representation and volume of data. Unlike single‐label ECG diagnosis, localisation is a multi‐class problem with potentially dozens of output classes (each corresponding to a distinct anatomical site or region). Obtaining enough training examples for each arrhythmia origin class is difficult; it demands collating data from many patients who have undergone successful ablations with known sites. To address small sample sizes, researchers have employed techniques such as data augmentation and transfer learning. In the WPW study, for instance, the model benefited from *multimodal transfer learning*; the CNN was pretrained on a large chest X‐ray data set (to learn cardiac silhouette features) and then fine‐tuned on the smaller WPW set, thereby improving accuracy with limited ECG samples [14]. This approach facilitated the incorporation of information on heart position and size without requiring thousands of WPW cases. Similarly, Zheng et al. achieved high accuracy, in part, by pooling multiple ECG recordings per patient, thereby expanding the training data and capturing beat‐to‐beat variability [8]. The use of one‐dimensional CNNs on raw ECG waveforms has been a crucial innovation, as these models can automatically learn waveform features (timing, voltage patterns) that differentiate origin sites [15], eliminating the need for manual feature engineering, which often misses subtle spatiotemporal cues.

Model generalisability is another challenge; a model trained in one centre may underperform in another due to differences in ECG machines, patient demographics, or the prevalence of certain arrhythmia types. To improve robustness, future models might incorporate physiology‐based augmentation (e.g., simulating ECGs from virtual heart models with varying orientations) or domain‐adaptation techniques to account for differences in ECG acquisition. Another innovation on the horizon is the use of *physics‐informed neural networks* or personalised cardiac models (digital twins) that integrate AI predictions with biophysical simulations of cardiac activation [16]. These could ensure that the AI's suggestions are physiologically plausible (e.g., predicting a site that leads to a QRS pattern matching the patient's ECG) and enhance clinician trust through more explainable outputs, such as predicted activation maps.

Model **i**nterpretability is indeed a significant technical hurdle. While deep learning models can be accurate, their “black‐box” nature may deter clinicians from fully trusting their outputs for high‐stakes decisions such as ablation target selection. Research is beginning to address this by providing explanations or visualisations of the AI's reasoning. For instance, attention mapping or gradient‐based methods can highlight which parts of the ECG trace most influenced the localisation decision [17, 18]. If an AI points to a PVC focus in the septum, showing that it keyed in on an atypical R‐wave pattern in lead V3 could provide reassurance and insight to the EP operator. Moreover, combining AI with well‐established physiological markers can be beneficial; one proposed hybrid model first uses deep learning to address inter‐subject variability, then applies more transparent algorithms for fine localisation [19]. Such hybrid or ensemble strategies aim to balance performance with interpretability. Technical innovation is also evident in the development of real‐time capable algorithms. In the EP lab, timing is crucial. Models must deliver predictions almost instantaneously upon detection of an arrhythmia. This has driven optimisations in model size and inference speed and has even led to the exploration of deploying AI within EP mapping systems or devices (on‐device AI) to enable immediate feedback [20, 21, 22]. Ensuring these algorithms can run quickly and reliably on standard hospital IT infrastructure (or directly integrated into pacing/mapping consoles) is a non‐trivial engineering task, requiring efficient code and validation against latency issues. Current AI‐ECG localisation models are designed to predict the site of earliest electrical activation and do not account for anatomical or procedural constraints, such as proximity to coronary arteries or regions that are not amenable to safe ablation. As such, algorithmic predictions must be interpreted in conjunction with imaging and intra‐procedural findings. Future multimodal approaches integrating ECG with anatomical imaging may help address this limitation and improve procedural planning [23].

Finally, data labelling and ground truth pose challenges. For supervised learning, one needs the true origin of each arrhythmia, which comes from invasive EP studies and ablations. Those can sometimes be difficult to interpret (e.g., multiple closely spaced foci or a diffuse substrate). Inconsistent or erroneous labels could confuse the model. To mitigate this, investigators have defined clear endpoint criteria (e.g., including only cases with unequivocal successful ablation at a site or using high‐density mapping to confirm the activation origin) [11]. Some have also reduced complexity by grouping anatomically adjacent sites into categories (e.g., the WPW model grouped 13 specific annular locations into four broader classes) to improve reliability [7]. As data sets grow, semi‐supervised approaches may enable the model to refine or relabel data by identifying consistent patterns across patients. Despite these challenges, the trajectory of innovation is promising. Each technical hurdle has prompted creative solutions, from multimodal learning to novel network architectures, pushing AI‐based ECG interpretation into realms once thought exclusive to invasive mapping. An emerging solution is to use foundation models and large‐scale, pretrained multimodal architectures. Foundation models trained on millions of ECGs, medical images, or combined physiologic signals can learn universal cardiac representations that are subsequently fine‐tuned for arrhythmia localisation tasks using relatively small, labelled EP data sets. Such models may substantially reduce sample size requirements, improve generalisability across institutions, and facilitate integration of ECG with complementary modalities such as chest X‐ray or cardiac imaging. As foundation models mature, their application to noninvasive arrhythmia source localisation is likely to accelerate translational adoption. Beyond supervised and self‐supervised learning, reinforcement learning may offer a future pathway for adaptive arrhythmia localisation. In this paradigm, an RL agent could treat the EP procedure as a sequential decision‐making process, in which each mapping action or catheter movement yields new electrical information and a corresponding reward signal based on proximity to the true arrhythmia source. Over time, the agent could learn policies that efficiently explore cardiac chambers, prioritise high‐yield regions, and converge on the site of origin with fewer mapping points. When combined with ECG‐based AI pre‐procedural localisation, RL could further refine intraprocedural targeting in real time, potentially reducing procedure duration while preserving accuracy.

### 2.5 Translational Research and Future Integration

Bridging the gap from bench to bedside is the next critical step for AI‐driven ECG localisation. Thus far, much of the evidence comes from retrospective analyses or single‐centre studies. Translational research efforts are underway to validate these AI tools prospectively and assess their impact on clinical decision‐making. One key direction is to conduct multicentre trials in which an AI localisation algorithm is prospectively used to guide ablation and to compare it with standard practice. Such studies would evaluate endpoints such as procedure time, success rate, and complication rate when AI assistance is enabled. The TAILORED‐AF trial (though focusing on atrial fibrillation mapping) provides a precedent, where a machine‐learning guided strategy improved ablation outcomes over conventional ablation [24]. For ventricular arrhythmias and WPW, similar trials could randomise patients to an AI‐informed mapping strategy versus usual care. Results from these would be key for regulatory approval and adoption. Early signals, like the improvement in outcomes noted by Fox et al. with AI‐informed mapping, justify the enthusiasm [6]. To ensure generalizability, these algorithms should ideally be tested in varied hospital settings and by operators with differing levels of experience. This will demonstrate that the AI can be a reliable co‐pilot across environments, not only in the one in which it was created.

For integration into EP workflows, it will be important that AI tools are user‐friendly and interoperable with existing systems. In practice, one could envision the following workflow: when an EP study is scheduled for, say, a VT ablation, the patient's ECG recordings (either a representative tachycardia 12‐lead or sinus rhythm ECGs that contain PVCs) are fed into the AI algorithm in advance. The algorithm outputs a probability map or ranking of likely origin sites. This information is presented to the electrophysiologist, perhaps as a graphical overlay on a heart diagram or an annotated 12‐lead ECG highlighting features of interest. Modern EP mapping systems could import this prediction; for example, the system might display a highlighted segment on the fluoroscopic or 3D map (e.g., “high likelihood focus in inferoseptal LV”). Such integration requires collaboration with device/software manufacturers to ensure regulatory compliance and seamless data flow. Notably, the interpretability and transparency of AI outputs will determine clinician acceptance. Therefore, part of integration is also educational, training EP clinicians to understand what the AI is (and isn't) telling them. Given the high stakes, these tools will likely be used confirmatory (to guide where to look first) rather than as an unquestioned truth. Over time, as confidence grows, AI recommendations may carry greater weight in decisions such as where to ablate or when to stop a procedure (e.g., if AI and initial ablation success align, one might reduce excessive substrate ablation).

Another future integration aspect is combining AI‐ECG localisation with other patient data to enhance accuracy, thereby moving toward multimodal AI in EP. We observed a hint of this with chest X‐ray integration; in the future, one could fuse ECG‐based predictions with imaging data, such as cardiac MRI scar maps or CT anatomical models. If an AI ECG algorithm suggests a focus in the outflow tract, and a pre‐procedure MRI shows scar in that region, the convergence of evidence strengthens the case and may even help fine‐tune the target (localise to scar border zone). Conversely, if modalities conflict, that may indicate a need for more thorough mapping. Thus, the AI might become one component of a larger decision‐support system. Wearable technology and continuous monitoring will also play a role [25]: future AI might analyse long‐term ECG or patch monitor data to catch spontaneous arrhythmias and localise them, so that by the time a patient sees an EP, there's rich information on what triggers the arrhythmia and where it comes from. Integrating continuous data analysis into clinical workflows (with alerts or reports) can improve the timing of interventions, enabling earlier treatment before arrhythmias become incessant or cause cardiomyopathy.

On the regulatory and implementation front, achieving FDA approval or CE marking for these AI algorithms will require demonstrating not only performance but also safety and bias mitigation. Models must be vetted for consistent accuracy across subgroups (e.g., does localisation accuracy hold in patients with prior infarcts vs. those without, or in women vs. men?). They should include robust cybersecurity and fail‐safe features. Experts have proposed a phased roadmap: starting with preclinical retrospective validation, then multicenter prospective studies, followed by regulatory review, and finally controlled roll‐out with post‐market surveillance to ensure continued efficacy [1]. We are currently in the early phase of this AI‐driven ECG localisation pathway. The next few years will likely see the first regulatory‐cleared tools in this domain if results remain positive.

## 3 Conclusion

AI applications in electrophysiology are moving beyond arrhythmia detection to therapy guidance. The specific use case of AI‐driven ECG interpretation for arrhythmia source localisation exemplifies this progression. By leveraging patterns in the 12‐lead ECG that humans alone cannot readily quantify, AI can noninvasively pinpoint the origin of an arrhythmia in the heart, effectively providing a map before the map. Recent studies in WPW and idiopathic ventricular arrhythmias have shown that such algorithms can achieve high accuracy and improve procedural efficiency. The clinical impact could be substantial: more targeted ablations, shorter procedure times, and improved patient outcomes. However, this innovation comes with challenges. Rigorous validation, integration into clinical systems, and maintenance of clinician confidence through interpretable outputs are essential steps before AI localisation becomes routine in EP practice. These developments highlight a key trend: the convergence of data science and electrophysiology expertise. As the field advances, electrophysiologists may soon collaborate as closely with data engineers as with imaging specialists, co‐designing AI tools that amplify clinical judgment rather than replace it. In a discipline defined by precision and timing, AI‐augmented ECG interpretation offers a novel means to enhance precision and save time. Embracing and critically evaluating this technology will be crucial for translating its promise into improved patient care in electrophysiology.

## Author Contributions

**Ibrahim Antoun:** conceptualization, methodology, validation, writing original manuscript. **Mahmoud Eldesouky, Alkassem Alkhayer, Ahmed Abdelrazik, Kaung Myat Thu, Mokhtar Ibrahim, Riyaz Somani, G. André Ng:** writing – review and editing.

## Funding

The authors received no specific funding for this work.

## Consent

The authors have nothing to report.

## Conflicts of Interest

The authors declare no conflicts of interest.

## Data Availability

The authors have nothing to report.
